# Supplementary material for: Merkel Cell Carcinoma—Update on Diagnosis, Management and Future Perspectives
Source: Cancers (Basel). 2022 Dec 23;15(1):103. doi: 10.3390/cancers15010103 (PMC9817518; doi:10.3390/cancers15010103)
Supplement: Supplementary file 1 [file cancers-15-00103-s001.zip › cancers-2079229-supplementary.pdf]

Review

Merkel Cell Carcinoma—Update on Diagnosis, Management and Future Perspectives

Table S1. Available active and/or recruiting and/or not yet recruiting, interventional clinical trials and expanded access programs (EAP) for combination therapies with checkpoint inhibitors for advanced unresectable or metastatic MCC available at https://clinicaltrials.gov/ as of April 2022. N: Number of patients for original estimated enrollment, RP2D: Recommended phase 2 dose, TLR: Toll like receptor, BORR: Best overall response rate, MTD: Maximum tolerated dose.

Available Clinical trials with Checkpoint Inhibitors in Combination Therapies for Advanced Unresectable or Metastatic MCC

NCT number	Phase	N	Drugs	Mode of action	Primary objective
**04393753**	2	40	- Domatinostat<br>- Avelumab in patients progressing under anti-PD1/PD-L1	- Histone deacetylase inhibitor<br>- Anti-PD-L1	ORR
**03787602**	1b/2	27	- KRT-232 +/-<br>- Avelumab in p53 Wild-Type MCC	- Oral inhibitor of MDM2, an E3 ubiquitin ligase<br>- Anti-PD-L1	RP2D of KRT-232 ORR
**04261855**	1b/2	65	- Avelumab + Lutenium-177-Dotatate<br>- Avelumab +<br>External Beam Radiation	- Anti-PD-L1<br>- Peptide receptor radionuclide therapy	PFS at 12 months
**04590781**	1b/2	142	- XmAb18087 +/-<br>- Pembrolizumab	- Monoclonal antibody targeting somatostatin receptor 2 and CD3<br>- Anti-PD-1	Treatment-emergent adverse events<br>ORR, CR, PR rate
**03901573**	1b/2a	64	- Atezolizumab<br>- NT-17	- Anti-PD-L1<br>- Efineptakin Alfa, a fusion protein of recombinant IL-7 and a hybrid Fc region	MTD, RP2D of NT-17
**02978625**	2	68	- Intralesional TVEC followed by TVEC and Nivolumab	- Oncolytic herpes virus immunotherapy<br>- Anti-PD-1	Response rate to TVEC alone<br>BORR to combination
**04916002**	2	268	- Intralesional CMP-001<br>-Cemiplimab-rwlc	- TLR9 agonist<br>- Anti-PD-1	ORR
**04116320**	1	32	- Focused US Ablation +/-<br>- Standard-of-care anti-PD1 +/-<br>- Imiquimod	- Continuous high-intensity focused US to ablate tissue<br>- Anti-PD-1<br>- TLR7/8 agonist	Safety of FUSA<br>Proportion of patients with increased CD8+ T cell tissue infiltration
**03747484**	1/2	16	- FH-MCVA2TCR<br>- Avelumab or Pembrolizumab,	- Autologous MCPyV-specific HLA-A02-restricted TCR-	Toxicity<br>BORR

			- Radiation therapy	transduced CD4+ and CD8+ T-cells<br>- Anti-PD-1	
**03435640**	1/2	393	- Intralesional NKTR-262<br>- Bempegaldesleukin<br>- +/-Nivolumab	- TLR7/8 agonist<br>- CD122 agonist<br>- Anti-PD-1	Safety and tolerability<br>ORR
**03000257**	1	118	- Venetoclax<br>- Rovzalpituzumab Tesirine<br>- ABBV-181	- BCL-2 inhibitor<br>- Antibody-drug conjugate directed against delta-like protein 3<br>- Anti-PD-1	Safety Pharmakokinetics of venetoclax and ABBV-181
**02635672**	1	50	- VIP152 with dose-escalation<br>- Pembrolizumab	- CDK9 inhibitor<br>- Anti-PD-1	Safety Pharmakokinetics<br>MTD, RP2D
**04458259**	1	37	- PF-07265807 +/-<br>- Sasanlimab	- Axl/mertk tumor-associated macrophage kinase inhibitor<br>- Anti-PD-1	Safety<br>ORR and CR rate
**04551885**	1	27	- FT516<br>- Avelumab	- CD16 Fc receptor<br>- Anti-PD-1	Dose-limiting toxicities
**05069935**	1	189	- FT538<br>- Avelumab	- Allogeneic NK-cell immunotherapy<br>- Anti-PD-L1	Safety<br>RP2D
**03841110**	1	64	- FT500 +/-<br>- ICI (nivolumab, pembrolizumab, atezolizumab) +/-<br>- IL-2	- Allogeneic, iPSC-derived NK cell immunotherapy<br>- ICI	Dose-limiting toxicity
**03228667**	2	105	- N803<br>- PD-1/PD-L1 inhibitors +/-<br>- PD-L1 t-haNK	- IL15 superagonist complex combined with BCG<br>- NK-92 expressing IL-2, CD16 and a CAR recognizing PD-L1	ORR
**04234113**	1/1b	96	- SO-C101 +/-<br>- Pembrolizumab	- IL15 superagonist<br>- Anti-PD-1	Safety<br>Dose-limiting toxicities
**03074513**	2	160	- Atezolizumab<br>- Bevacizumab	- Anti-PD-L1<br>- Anti-VEGF	ORR
**04902040**	1b/2	12	- Radiation therapy<br>- PD-1/PD-L1 inhibitors +/-<br>- Plinabulin<br>after progression on anti-PD-(L)1	- Radiation therapy<br>- PD-1/PD-L1 inhibitors +/-<br>- Polymerization of tubulin blockade	Safety<br>ORR
**05269381**	1	36	-Cyclophosphamide -<br>Neoantigen peptide vaccine<br>- Sargramostim<br>- Pembrolizumab	- Chemotherapy<br>- Vaccine<br>- GM-CSF<br>- Anti-PD-1	Safety
**03236935**	1b	12	- L-NMMA<br>- Pembrolizumab	- NG-monomethyl-L-arginine, a pan-nitric oxide synthase inhibitor<br>- Anti-PD-1	MTD
**04725331**	1/2a	48	- Intralesional BT-001<br>- Pembrolizumab	- Oncolytic Vaccinia virus encoding for human CTLA4-specific antibody 4-E03 and human GM-CSF<br>- Anti-PD-1	Safety<br>RP2D<br>ORR<br>Disease control rate

**05086692**	1/2	80	- MDNA11 +/-<br>- Anti-PD1 or Anti-PD-L1	- Long-acting "beta-only" recombinant interleukin-2 (rIL-2) albumin fusion<br>- Anti-PD1 or Anti-PD-L1	Safety<br>RP2D

Table S2. Available active and/or recruiting and/or not yet recruiting, interventional clinical trials and expanded access programs (EAP) for checkpoint inhibitors in advanced unresectable or metastatic MCC available at https://clinicaltrials.gov/ as of April 2022. N: Number of patients for original estimated enrollment, RP2D: Recommended phase 2 dose, EAP: expanded access program, TLR: Toll like receptor, MTD: Maximum tolerated dose.

Available Clinical Trials with Checkpoint Inhibitors in Advanced Unresectable or Metastatic MCC

NCT number	Phase	N	Drugs	Mode of action	Primary objective
**03783078**	3	50	- Pembrolizumab as first-line agent	- Anti-PD-1	ORR
**04792073**	2	36	- Avelumab<br>- Comprehensive Ablative Radiotherapy	- Anti-PD-L1	PFS
**03599713**	2	90	- Retifanlimab	- Anti-PD-1	ORR
**03089658**	EAP		- Avelumab	- Anti-PD-L1	Available results [15]
**03071406**	2	50	- Nivolumab<br>- Ipilimumab<br>- +/- Stereotactic body radiation	- Anti-PD-1<br>- Anti-CTLA4	ORR<br>Preliminary results available [102]
**02155647 JAVELIN Merkel 200**	2	84	- Avelumab<br>Part A: after chemotherapy failure<br>Part B: first-line	- Anti-PD-L1	Part A: BORR<br>Part B: Durable response rate<br>Available results [7, 9, 11]
**03304639**	2	96	- Pembrolizumab +/-<br>- Stereotactic body radiation	- Anti-PD-1	PFS
**03071757**	1	180	- ABBV-368<br>+/-ABBV-181	- Anti-OX40 agonist<br>- Anti-PD-1	Safety Pharmakokinetics of ABBV-368
**03212404**	1	80	- Cosibelimab	- Anti-PD-1	Dose-limiting toxicity<br>Safety<br>ORR
**04187872**	1	15	- Laser Interstitial Thermal Therapy<br>- Pembrolizumab in patients with brain metastasis after failure of stereotactic therapy	- Minimally invasive neurosurgical technique<br>- Anti-PD-1	Immune effect of combination therapy
**04260802**	1b/2a	80	- OC-001 +/-<br>- Anti-PD1 or Anti-PD-L1	- Targets tumor necrosis factor receptor<br>- Anti-PD1 or Anti-PD-L1	Dose-limiting toxicity<br>Safety
**04157985**	3	578	Treatment duration of PD-1/PD-L1 inhibitors among responders	- Anti-PD-1/PD-L1	PFS
**04140526**	1a/2b	91	- ONC-392 +/-<br>- Pembrolizumab	- Humanized anti-CTLA4<br>- Anti-PD-1	MTD, RP2D<br>Dose-limiting toxicity<br>Safety
**05078047**	3	646	Evaluation of a reduced dose intensity among responders to ICI	- ICI	PFS

Table S3. Available active and/or recruiting and/or not yet recruiting, interventional clinical trials and expanded access programs (EAP) for targeted therapies and immune based strategies in advanced unresectable or metastatic MCC available at https://clinicaltrials.gov/ as of April 2022. N: Number of patients for original estimated enrollment, RP2D: Recommended phase 2 dose, EAP: expanded access program, TLR: Toll like receptor, MTD: Maximum tolerated dose, TIL: Tumor infiltrating lymphocytes.

Available Clinical Trials with Targeted Therapies and Immune-based Strategies in Advanced Unresectable or Metastatic MCC

NCT number	Phase	N	Drugs	Mode of action	Primary objective
**02465957**	2	24	- aNK<br>- ALT-803	- Activated NK-92 (allogenic Natural Killer cell line)<br>- IL-15 superagonist complex	PFS
**02036476**	2	12	- Cabozantinib after platinum-based therapy progression	- Multi-target tyrosine kinase inhibitors	Disease Control Rate, Available results [97]
**02819843**	2	34	- T-VEC +/-<br>- Hypofractionated radiotherapy for skin metastasis	- Oncolytic herpes virus immunotherapy	CR and PR rate
**04160065**	1	20	- Intralesional IFx-Hu2.0 for skin, LN metastasis	- Plasmid DNA vaccine encoding for a streptococcal membrane protein, Emm55	Treatment-emergent adverse events
**04349436**	1b/2	30	- Intralesional RP1 in organ transplant recipients for skin, LN metastasis	- Oncolytic herpes virus 1	Adverse events<br>ORR
**05120271**	1/2	98	- BOXR1030<br>- Lymphodepleting chemotherapy for GPC3+ MCC	- GPC3-targeted CAR T Cell therapy	Treatment-emergent adverse events, MTD, Dose-limiting toxicity
**04853602**	EAP		- Intralesional IFx-Hu2.0 for skin, LN metastasis	- Plasmid DNA vaccine encoding for Emm55	
**04047251**	2	96	- FF-10850 Topotecan Liposome Injection	- A novel liposomal form of Topotecan	Dose-limiting toxicity, Adverse events, MTD, RP2D
**05267626**	1/2	126	- AU-007	- Anti-IL-2 antibody inhibiting IL-2Ra binding	Dose-limiting toxicity, Adverse events, MTD, RP2D
**03544723**	2	40	- Intralesional Adp53<br>- ICI	Adenoviral p53 gene therapy	ORR<br>Safety
**04276597**	2	50	- 177Lu-DOTATOC	- 177Lu labeled somatostatin receptors targeting ligand	ORR

**02479698**	2	30	- BK-CTLs	- Allogeneic, HLA-matched BK-specific cytotoxic T-lymphocytes	Safety<br>Incidence of GVHD<br>Response rate
**04246671**	1	45	- TAEK-VAC-HerBy vaccine	- Therapeutic vaccine	Dose-limiting toxicity
**04242199**	1	140	- INCB099280	- Targets microsatellite instability-high/mismatch repair deficient tumors	Treatment-emergent adverse events
**04272034**	1	140	- INCB099318	- Targets microsatellite instability-high/mismatch repair deficient tumors	Treatment-emergent adverse events
**04596033**	1	24	- GEN-011<br>- IL-2 +/-<br>- Lymphodepletion (fludarabine, cyclophosphamide)	- Adoptive T cell therapy with identified immunogenic neoantigens from each tumor	Safety
**03935893**	2	240	- Lymphodepletion<br>- Autologous TIL<br>- IL-2	- Adoptive transfer of autologous TIL	ORR
**05076760**	1	18	- Intralesional MEM-282 with expected abscopal effect	- Oncolytic adenovirus encoding for IFNb and CD40-ligand	MTD<br>Safety
